# Patients’ perspectives on video consultation for non-communicable diseases: a qualitative study in Singapore

**DOI:** 10.3399/BJGPO.2023.0103

**Published:** 2023-12-13

**Authors:** Mui Suan Tan, Gary Chun-Yun Kang, Rodney Jin Kai Fong, Nian Kai Cheong, Haixiao Shi, Ngiap Chuan Tan

**Affiliations:** 1 SingHealth Polyclinics, Singapore; 2 SingHealth Duke-NUS Family Medicine Academic Clinical Programme, Singapore; 3 Lee Kong Chian School of Medicine, Nanyang Technological University, Singapore

**Keywords:** health information technology acceptance model, non-communicable diseases, primary health care, telemedicine, video consultation

## Abstract

**Background:**

The COVID-19 pandemic increased the use of telemedicine in primary care services. Understanding patients’ perspectives on telemedicine is pivotal for its wider adoption in managing non-communicable diseases (NCDs) in the community.

**Aim:**

To explore the views and concerns of patients who have yet to use video consultation (VC) for NCD management in Singapore.

**Design & setting:**

This qualitative study was conducted in a primary care clinic in Singapore.

**Method:**

In total, 16 patients participated in individual in-depth interviews. The participants had type 2 diabetes mellitus and/or hypertension and/or hyperlipidaemia without prior VC experience. They were purposively enrolled in the polyclinic. Audited transcripts were independently coded by two investigators. Thematic analysis was performed to identify perspectives on telemedicine based on the health, information, and technology zones of the Health Information Technology Acceptance Model.

**Results:**

The following three themes emerged: perceived benefits of VC utility; perceived barriers of VC adoption; and potential challenges of VC. Participants viewed VC as safe and convenient if they had stable NCD. They voiced concerns on possible suboptimal care owing to the absence of physical examination, network connectivity, and personal medical data security. Participants highlighted challenges of VC uptake such as digital health familiarity, availability of their own mobile and telemonitoring devices, and healthcare costs consideration.

**Conclusion:**

Addressing the concerns and challenges highlighted by non-VC users can help physicians and policymakers explore ways to scale up telemedicine in Singapore. A hybrid clinical care model comprising in-person visits and VC may be the way forward for NCD management.

## How this fits in

Understanding patients’ perspectives on telemedicine is essential for its wider adoption and development in post-pandemic primary care. This study found that non-video consultation (VC) users viewed VC as safe and convenient if they had stable non-communicable disease (NCD). They voiced concerns on possible suboptimal care owing to the absence of physical examination. Combining in-person visits and teleconsultation may be a desirable hybrid care model.

## Introduction

Telemedicine is the systemic provision of healthcare services via information and communications technology, covering teletreatment, telemonitoring, and telesupport.^1^ Teleconsultation was increasingly adopted to provide chronic, primary, and specialty care during the COVID-19 pandemic.^2^ Teleconsultation converts a face-to-face interaction between doctors and patients to a virtual consultation, through a phone call or video call. Patients can remain in the comfort of their homes for review of their medical problems.

Telemedicine is set to become a key feature of Singapore’s healthcare landscape with an ageing population,^3,4^ in order to cope with the expected rise in the number of patients with NCDs such as type 2 diabetes mellitus (T2DM). Singapore’s Ministry of Health (MOH) has set up National Telemedicine Guidelines and an online course to guide doctors on telemedicine services.^3^ Local primary care clinics have introduced teleconsultations to patients.^5^

International studies have provided perspectives on the utility and delivery of telemedicine.^6–11^ About three-quarters of patients in South Korea preferred teleconsultation, and their acceptance of teleconsultation varied according to age, education, and income.^6^ However, they were concerned about reliability, cost, and technical difficulties. In a further study in the US,^7^ patients cited convenience, privacy, and comfort as important considerations.

VCs are considered superior to telephone consultations. In a British qualitative study in primary care, VCs provided better visual cues, rapport, and communication.^8^ These visual cues allowed for a formal and focused consultation. However, if physical examinations were necessary, patients preferred face-to-face consultations.^12^

VC conducted via Zoom Video Communications was introduced in Singapore in mid-2020 for patients with stable NCDs.^13^ The preference, acceptance, and concerns of local patients towards teleconsultation is unknown. A survey on patients with NCDs showed that only half were willing to use telemonitoring, which complement telemedicine.^14^ Factors affecting their willingness included technological literacy, beliefs, and previous technology utility.

As VC is relatively new to the local population, future expansion of the service will require knowledge of how receptive patients are towards VC, especially for those who have not adopted VC. This study aimed to explore the views and concerns of multi-ethnic Asian patients with T2DM and/or hypertension and/or hyperlipidaemia, who are non-users of VC, towards VC for NCD management in primary care.

## Method

### Study design

This study employed a qualitative research method. Individual in-depth interviews (IDIs) were organised to understand participants’ subjective and multidimensional views and concerns towards VC, aided by a video and interview guide.

### Conceptual framework

A conceptual framework helps to piece together the multidimensional perspectives of a topic to facilitate understanding of their inter-relationship. Such a framework supports the design of the interview guide in this study (see Supplementary Appendix S1). The Health Information Technology Acceptance Model (HITAM)^15^ assessed consumers’ acceptance of technology implementation in health care, and was developed as an extension of the Technology Acceptance Model and the Health Belief Model. Validated in South Korea to describe health consumers’ behavioural intention, the framework categorised factors into ‘health’, ‘information’, and ‘technology’ zones. The health information technology (HIT) of interest in this study was VC. The framework was selected to focus on a tech-enabled modality of telemedicine delivery and utility. It includes 1) the health zone on views about participants’ health status, beliefs, and concerns; 2) information zone on normative beliefs; and 3) technology zone on HIT reliability and self-efficacy.

### Study site and period of study

SingHealth Polyclinics is a public primary care institution with nine polyclinics serving >1.3 million residents in Singapore. This study was conducted from March–August 2021 in Sengkang Polyclinic, located in north-east Singapore, which managed >900 patients daily, including those with NCDs.

### Study population

The target participants were patients on follow-up at the polyclinic for T2DM and/or hypertension and/or hyperlipidaemia, and non-users of VC at the point of recruitment. They included Asian adults aged ≥21 years, of any sex or ethnic group, and were able to understand and communicate in English. Those with any disability that rendered them unable to provide informed written consent or unable to speak or understand English were excluded.

### Recruitment

Potential participants were invited in-person during their clinic visit, and the reasons for doing the study were explained to them. Participation was voluntary without any impact on their care. Purposive sampling was conducted to include participants of different ages and education. Their demographic profiles were postulated to influence their views on VC. Informed written consent was obtained from all participants before the interview in a private room.

### Interviews and transcribing

The participants underwent one-to-one interviews conducted by the lead investigator, a family physician trained in qualitative research. The interviews were organised individually at the study site for participants to share their personal views of VC frankly. Demographic data were collated using the participant demographics questionnaire. A video demonstrating VC in primary care was shown to participants, followed by a semi-structured interview aided by the topic guide. Interview notes were taken. All participants were reimbursed with grocery store vouchers of 20 SGD (approximately 15 USD; 12.50 GBP) as tokens of appreciation for their time. The interviews were audiorecorded and transcribed verbatim by a professional transcriber. The study team members audited the transcripts against the recordings to ensure accuracy.

### Coding and analysis

Data were analysed according to thematic analysis,^16^ and executed iteratively after each interview. Two investigators read the full transcripts independently to generate initial ideas, before deriving an initial coding frame. Regular meetings were held to discuss and modify the coding frame for data analysis. Perspectives were cross-checked and clarified with participants during subsequent IDIs. The mutually agreed codes were systematically assembled into a final coding frame. Any difference or disagreement in coding was deliberated between the investigators to reach a consensus. Data saturation was considered at the point when no new code emerged from further interviews. The codes were then analysed iteratively to identify emergent themes, and framed against the HITAM zones. Additional perspectives were grouped if they did not align to the zones. NVivo (version 12) software was used to support data analysis.

## Results

In total, 18 people were approached and 16 agreed to participate. The participants included seven males and nine females, and their ages ranged from 38–65 years (mean age 54.5 years). The interviews lasted between 16 and 28 minutes. Table 1 shows the demographic characteristics of the participants.

**Table 1.** Demographic characteristics of study participants (*N* = 16)

Characteristic	*n* (%)
Sex	
Male	7 (44)
Female	9 (56)
Age, years	
<50	5 (31)
50–60	5 (31)
>60	6 (38)
Ethnic group	
Chinese	11 (69)
Malay	2 (13)
Indian	2 (13)
Ceylonese	1 (6)
Highest education level	
Primary	6 (38)
Secondary	2 (13)
Diploma or high school	5 (31)
University	3 (19)
Type of housing	
1–3 room public housing apartment	2 (13)
4–5 room public housing apartment	12 (75)
Condominium or private property	2 (13)
Need to pay tax last year?	
Yes	9 (56)
No	7 (44)
Received medical subsidy?	
Yes	6 (38)
No	10 (63)
NCDs	
T2DM	10 (63)
HTN	10 (63)
HLD	15 (94)
T2DM + HTN + HLD	7 (44)
Others^a^	6 (38)
Total number of NCDs	
≥4	3 (19)
3	8 (50)
1 or 2	5 (31)

^a^Others included obesity, gout, osteoarthritis, and hypothyroidism. HLD = hyperlipidaemia. HTN = hypertension. NCD = non-communicable disease. T2DM = type 2 diabetes mellitus.

Results relating to the participants’ views and concerns of VC were grouped into the following three main themes: perceived benefits of VC utility; perceived barriers of VC adoption; and potential challenges of VC. Themes and subthemes were supported with corresponding verbatim.

### Perceived benefits of VC utility

#### Stable NCDs were suitable for VC

Participants with stable NCDs viewed themselves as suitable for VC as they expected minimal changes to their current treatment plans:

*’I feel* [the] *general public will benefit from this, and chronic illness people like me.’* (P014, aged 47 years, male, degree-holder, employee with well-controlled T2DM, hypertension, hyperlipidaemia, and osteoarthritis)

#### Perceived safe environment

Participants viewed VC at home as safe for them during the COVID-19 pandemic as they could avoid polyclinics, which also manage patients with acute respiratory infections:

*’I think it’s a very good practice … no need to go directly in contact with another person … the separation is there.’* (P004, aged 65 years, male, high school education, employee with hypertension and hyperlipidaemia)

*’A lot of things changed already because of this COVID-19 … you have to have safe distancing.’* (P001, aged 63 years, male, high school education and receiving medical subsidy, employee with hyperlipidaemia)

#### Convenience and time-savings

Participants perceived VC as convenient owing to time-savings from travelling and waiting in clinics, and cost-saving resulting from not having to take a day off work:

*’Raining I still need to make it here* [clinic]*, whereas for VC, rain or shine, the doctor is there … it is a very beneficial thing for patients.’* (P006, aged 63 years, female, diploma-holder living in private property, employee with hypertension)

*’When I come down to the clinic, I actually take leave instead of taking time off for all these visits. If we have VC right now, it’s working from home. I can just pop by and have a VC done.’* (P015, aged 38 years, female, degree-holder, office-worker with T2DM and hyperlipidaemia)

### Perceived barriers of VC adoption

#### Concern about suboptimal care

Participants were concerned that the lack of physical examination in VC might affect clinical care. A clinic visit might be necessary if they needed investigations or thorough assessment:

*’The few days I got leg pain, backache, all these I cannot talk* [mention] *in the video … My leg swollen they* [doctors] *also cannot see. Blood test we have to come here.’* (P005, aged 61 years, female, primary school education, cleaner with T2DM, hyperlipidaemia, and osteoarthritis)

*’I think the perception is that patients still need the physical examination by the doctor to see the actual cause of the illness.’* (P011, aged 52 years, male, diploma-holder, employee with T2DM, hypertension, and hyperlipidaemia)

#### Concern about medication supply

In a face-to-face consultation, medication collection is usually done on the same day in the clinic. As it takes days to deliver medications to patients after VC, participants highlighted the need to ensure adequate medications at home:

*’I’m OK with VC, as long as I have sufficient medication while waiting for the medicine to come.’* (P015, aged 38 years, female, degree-holder, office-worker with T2DM and hyperlipidaemia)

#### Network connectivity and personal medical data security

Some participants were concerned about network connectivity:

*’Some families’ internet connection may not be good …* [upcoming 5G network] *it’s on the infrastructure … Now 4G* [is] *also very stable.’* (P011, aged 52 years, male, diploma-holder, employee with T2DM, hypertension, and hyperlipidaemia)

Most believed that data would be managed securely and kept confidential:

*’Personally, I feel it’s quite secured … Zoom meetings are secured data over internet … you don’t repeat the meeting.’* (P014, aged 47 years, male, degree-holder, employee with well-controlled T2DM, hypertension, hyperlipidaemia, and osteoarthritis)

### Potential challenges of VC

#### Digital health familiarity

Participants competent in IT skills were confident of VC and did not expect technical difficulties:

*’We’re all working from home. I think Zoom is fine. Because I’ve tried using Zoom for video conferencing.’* (P015, aged 38 years, female, degree-holder, office-worker with T2DM and hyperlipidaemia)

However, less tech-savvy participants were concerned and anticipated help from their family:

*’I’m not IT savvy … I have to learn and catch up with all the advanced technology.’* (P001, aged 63 years, male, high school education and receiving medical subsidy, employee with hyperlipidaemia)

*’Computer I’m not sure* [familiar]*, how can I catch up? That’s why I go to SG Digital* [Singapore Digital: aims to equip Singapore’s older adults with digital skills]*.’* (P002, aged 56 years, female, primary school education, unemployed with T2DM and hyperlipidaemia)

#### Availability of own mobile and telemonitoring devices

In order to be eligible for VC, patients need to have their own mobile devices to join the session, and a blood pressure (BP) machine to report their BP to the doctor. Participants did not foresee difficulties with VC if they owned these devices; however, those without the devices might encounter challenges with VC:

*’I’ve got no issue because I got all these machines* [BP machine and glucometer] *at home. But some* [low-income patients] *really can’t monitor themselves at home regularly. They have to go to the polyclinics or the GP, the clinic itself.’* (P013, aged 43 years, female, diploma-holder, employee with T2DM, hypertension, and hyperlipidaemia)

#### Healthcare expenses

Direct and indirect cost considerations were brought up by participants. They believed that VC should be made affordable:

*’It doesn’t make sense that the price is more expensive; I might as well see my doctor* [in-person]*.’* (P006, aged 63 years, female, diploma-holder living in private property, employee with hypertension)

*’I can’t really say how much it should be but the main issue is that everybody can afford it.’* (P013, aged 43 years, female, diploma-holder, employee with T2DM, hypertension, and hyperlipidaemia)

Other considerations include the cost of Wi-Fi, mobile data, and cost savings in terms of time and travelling:

*’The internet — every month I have to pay, then I can use it at home.’* (P003, aged 62 years, female, primary school education receiving medical subsidy and living in public housing, homemaker with T2DM, hypertension, and hyperlipidaemia)

*’The government has to make it very attractive … it has to be low cost. People will certainly think "why do I need to spend more when I’m using my time to set up my gadget".’* (P011, aged 52 years, male, diploma-holder, employee with T2DM, hypertension, and hyperlipidaemia)

## Discussion

### Summary

This qualitative study referred to the HITAM model as a conceptual framework to understand non-VC users’ views and concerns towards VC for NCD management in primary care in Singapore. This study identified the following three themes: 1) perceived benefits of VC utility; 2) perceived barriers of VC adoption; and 3) potential challenges of VC.

### Strengths and limitations

This study used the HITAM model as a framework to explore participants’ views and concerns towards VC, which they had yet to try. This novel approach helped to showcase how health, information, and technology factors might influence VC adoption among patients with NCDs. Addressing the factors would allow physicians to explore ways to encourage VC uptake among primary care patients with NCDs in future.

This study had its limitations. Only English-speaking participants were included. Participants were introduced to VC via video and not direct experience. However, interviewing these non-users was useful for exploring reasons why they had yet to adopt VC. The NCDs included in the study generally do not require physical examination by clinicians during follow-up care. Thus, the findings cannot be generalised to other NCDs requiring physical examination such as asthma and chronic obstructive pulmonary disease. Future research could be done to include these respiratory NCDs, and explore the experience of the various stakeholders in the VC system, including VC users with NCDs and healthcare providers.

### Comparison with existing literature

Participants with stable NCDs were keen to adopt VC, but had concerns about clinical care. A British study showed that while patients appreciated the ‘customer-care’ aspect of enhanced convenience in teleconsultation, they prioritised the ‘clinical care’ of doctor–patient interaction.^12^ King *et al* showed how telemedicine could enhance face-to-face services based on patients’ needs and context.^17^ Proper patient selection and a hybrid clinical care model comprising VC and face-to-face consultations could be the way forward.^7,18^ Doctors can identify suitable patients in clinic and offer them VC for subsequent reviews.

### Implications for practice

This study was conducted in the midst of the pandemic, when VC was newly introduced. Various countries envisioned family physicians adopting digital technology advancements to care for the population.^18,19^ Telemedicine would be scaled up for home-based, technology-enabled self-management of NCDs to improve primary care capability.^20,21^ However, a concern raised in the present study was the availability of telemonitoring devices needed for VC. To complement VC for NCD follow up, provision of home telemonitoring devices and do-it-yourself investigations for patients can be explored. A Singapore study showed that telemonitoring with teleconsultation improved BP control and was cost-effective.^22^ Self-administered laboratory tests for NCD might also be feasible.^23^ These methods could encourage and support patients with NCD to opt for VC.

It is important to recognise VC as a system involving the patient and other stakeholders. Healthcare costs and network connectivity highlighted in the study can also influence their willingness to adopt VC. The PERCS (Planning and Evaluating Remote Consultation Services) framework by Greenhalgh *et al* has depicted how various domains, such as the patient, staff, organisation, technologies, and the wider system, interact and influence one another.^24^ Gilbert *et al* reported that VC implementation posed challenges such as the lack of suitable infrastructure, and a fully integrated system would require infrastructural improvement.^17,25^ As demonstrated in a Scotland case study,^26^ a well-established infrastructure supported by a national strategic vision can help to expand VC services in times of need. Addressing the concerns and challenges identified in this study will allow physicians and policymakers to review the resources and shift mindsets to encourage VC uptake. The MOH Office for Healthcare Transformation was set up to reshape Singapore’s health system and explore how technologies can be incorporated in primary care. In addition, Singapore’s Infocomm Media Development Authority is developing a 5G network coverage,^27^ which will strengthen the technological infrastructure for VC. These concerted efforts are good initial steps to scale up telemedicine in Singapore.

In conclusion, this study used the HITAM model to explore non-VC users’ perspectives towards adopting VC for NCD follow up. Participants viewed VC as convenient and safe. Concerns about the absence of physical examination and medication supply were perceived barriers. Challenges to VC adoption included digital health unfamiliarity and healthcare expenses. A hybrid clinical care model comprising in-person and teleconsultation may be the way forward for NCD management.

## Data

Data that support the findings of this study are available from the corresponding author on reasonable request.
